# A mixed adenoneuroendocrine carcinoma of the pancreas: a case report

**DOI:** 10.1186/s40792-016-0263-1

**Published:** 2016-11-15

**Authors:** Kouki Imaoka, Saburo Fukuda, Hirofumi Tazawa, Yoshio Kuga, Tetsuya Mochizuki, Yuzo Hirata, Seiji Fujisaki, Mamoru Takahashi, Toshihiro Nishida, Hideto Sakimoto

**Affiliations:** 1 Department of Surgery, Chugoku Rousai Hospital, 1-5-1 Hirotagaya, Kure, Hiroshima, 737-0193 Japan; 2 Department of Gastroenterology, Chugoku Rousai Hospital, Kure, Japan; 3 Department of Diagnostic Pathology, Chugoku Rousai Hospital, Hiroshima, Japan; 4 Faculty of Medicine, Hiroshima University, 1-2-3 Kasumi, Hiroshima, 734-8551 Japan

## Abstract

A tumor consisting of an adenocarcinoma component and a neuroendocrine carcinoma component, with each component accounting for at least 30% of the tumor, is defined as a mixed adenoneuroendocrine carcinoma (MANEC). We report a case of MANEC of the pancreas. A 63-year-old man presented with hyperglycemia and was referred to our hospital for further examination. Abdominal contrast-enhanced computed tomography (CT) revealed a mass of 2 cm in size in the pancreas head with portal vein narrowing. Fluorin-18 fluorodeoxyglucose-positron emission tomography (FDG-PET) CT revealed increased accumulation in the mass of the pancreas head. Endoscopic retrograde cholangiopancreatography (ERCP) showed severe narrowing of the main pancreatic duct. Cytological analysis by endoscopic ultrasonography-guided fine-needle aspiration (EUS-FNA) suggested a neuroendocrine tumor. Under the diagnosis of neuroendocrine tumor, pancreaticoduodenectomy with portal vein resection and regional lymph node dissection was performed with curative intent. Histological examination revealed that the tumor consisted of two cell populations. One was well- to moderately differentiated tubular adenocarcinoma. This cell component accounted for 45% of the whole tumor. The second component was non-adenocarcinoma cells arranged in a nest, and the cells had round nuclei, abundant cytoplasm, and coarse chromatin. The Ki67 labeling index was 40%. Immunohistochemically, the adenocarcinoma cells were positive for CEA but negative for chromogranin A (CgA) and synaptophysin (Syn), while the non-adenocarcinoma cells were positive for the expression of CgA and Syn but negative for CEA. Based on the findings, a diagnosis of MANEC of the pancreas was made. Postoperatively, lymph node metastasis and peritoneal dissemination developed rapidly and he died the 6 months after the operation. Due to the small number of reported cases of MANEC of the pancreas, its clinical behavior remains unclear and a standardized management protocol has not been established. Further investigation of more cases of this rare entity is necessary.

## Background

In the 2010 World Health Organization (WHO) classification of neuroendocrine neoplasms in the digestive system [1], tumors consisting of an adenocarcinoma component and a neuroendocrine carcinoma component, in which each component accounts for at least 30% of the tumor, are defined as mixed adenoneuroendocrine carcinomas (MANECs) [1]. MANECs can occur in various organs including the gallbladder [2], bile duct [3], stomach [4], colon [5], and cecum [6]. This classification also applies to pancreatic neuroendocrine neoplasms. However, MANEC located in the pancreas is extremely rare. Herein, we report a case of MANEC of the pancreas and present a brief literature review.

## Case presentation

A 63-year-old man presented with hyperglycemia and was referred to our hospital for further examination in April 2015. He had no past history including pancreatic disorders. Laboratory tests showed the following: pancreatic amylase, 291 IU/l (normal range, 40–129 IU/l); BS, 219 mg/dl (70–109 mg/dl); and HbA1c, 7.5% (4.6–6.2%). Serum level of the tumor marker carcinoembryonic antigen (CEA), 4.7 ng/ml, was within normal range (<5.0 ng/ml), while serum levels of the tumor markers carbohydrate antigen 19-9 (CA19-9), 51.1 U/ml (<37 U/ml), DUPAN-2, 53 U/ml (<25 U/ml), and SPAN-1, 45.9 U/ml (<10 U/ml), were slightly elevated.

**Fig. 1:**
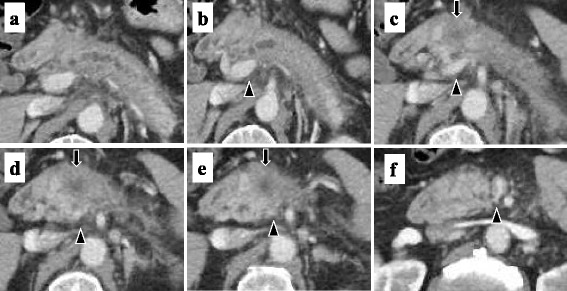
Abdominal contrast-enhanced computed tomography (CT) showed diffuse enlargement of the pancreas with increased CT level in the peri-pancreatic fatty tissue and revealed a mass of 2 cm in size in the pancreas head (**c–d**, *arrow*). The mass was poorly enhanced in the arterial phase and was gradually enhanced in the venous phase. The portal vein showed narrowing (**b–f**, *arrowhead*)

**Fig. 2:**
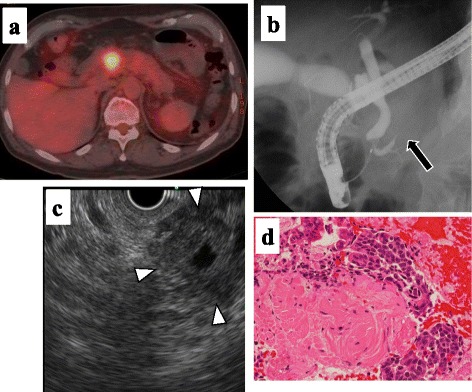
PET-CT revealed increased accumulation in the mass of the pancreas head (maximum standardized uptake value (SUV max): 5.6 (initial) and 7.8 (delayed) (**a**). Endoscopic retrograde cholangiopancreatography (ERCP) showed severe narrowing and obstruction of the main pancreatic duct by the tumor (*arrow*) (**b**). Endoscopic ultrasonography (EUS) showed the tumor as a low echoic mass (**c**). Cytological analysis by means of EUS-guided fine-needle aspiration (FNA) suggested a neuroendocrine tumor (G2) (**d**)

Abdominal contrast-enhanced computed tomography (CT) showed diffuse enlargement of the pancreas with increased CT level in peri-pancreatic fatty tissue and revealed a mass of 2 cm in size in the pancreas head. The mass was poorly enhanced in the arterial phase and was gradually enhanced in the venous phase. The portal vein showed narrowing, suggestive of tumor invasion (Fig. 1). FDG-PET CT revealed increased accumulation in the mass of the pancreas head (Fig. 2a). Endoscopic retrograde cholangiopancreatography (ERCP) showed severe narrowing of the main pancreatic duct (Fig. 2b). Cytology of pancreatic juice collecting during the ERCP did not reveal malignant cells. Cytological analysis by means of endoscopic ultrasonography-guided fine-needle aspiration (EUS-FNA) suggested a neuroendocrine tumor (G2) (Fig. 2c, d).

**Fig. 3:**
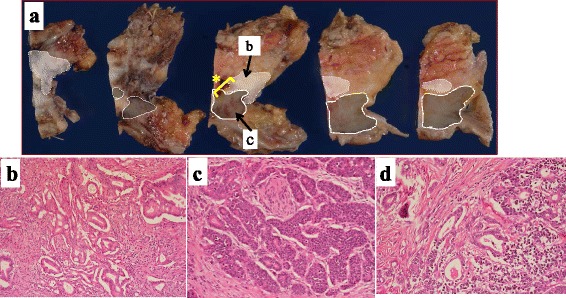
Macroscopically, a 2-cm-sized mass in the pancreas head was presented as the resected specimen. Histological examination revealed that the tumor consisted of two cell populations. The *white-shaded area* indicates a component of well- to moderately differentiated tubular adenocarcinoma cells and the *gray-shaded area* indicates a component of non-adenocarcinoma cells (**a**). The adenocarcinoma cells were arranged in an irregular pattern (**b**). Non-adenocarcinoma cells are arranged in a nest, and the cells had round nuclei, abundant cytoplasm, and coarse chromatin (**c**). An intermixed central zone exists between the two cell components (**d**) (*asterisk* part in Fig. 3a)

**Fig. 4:**
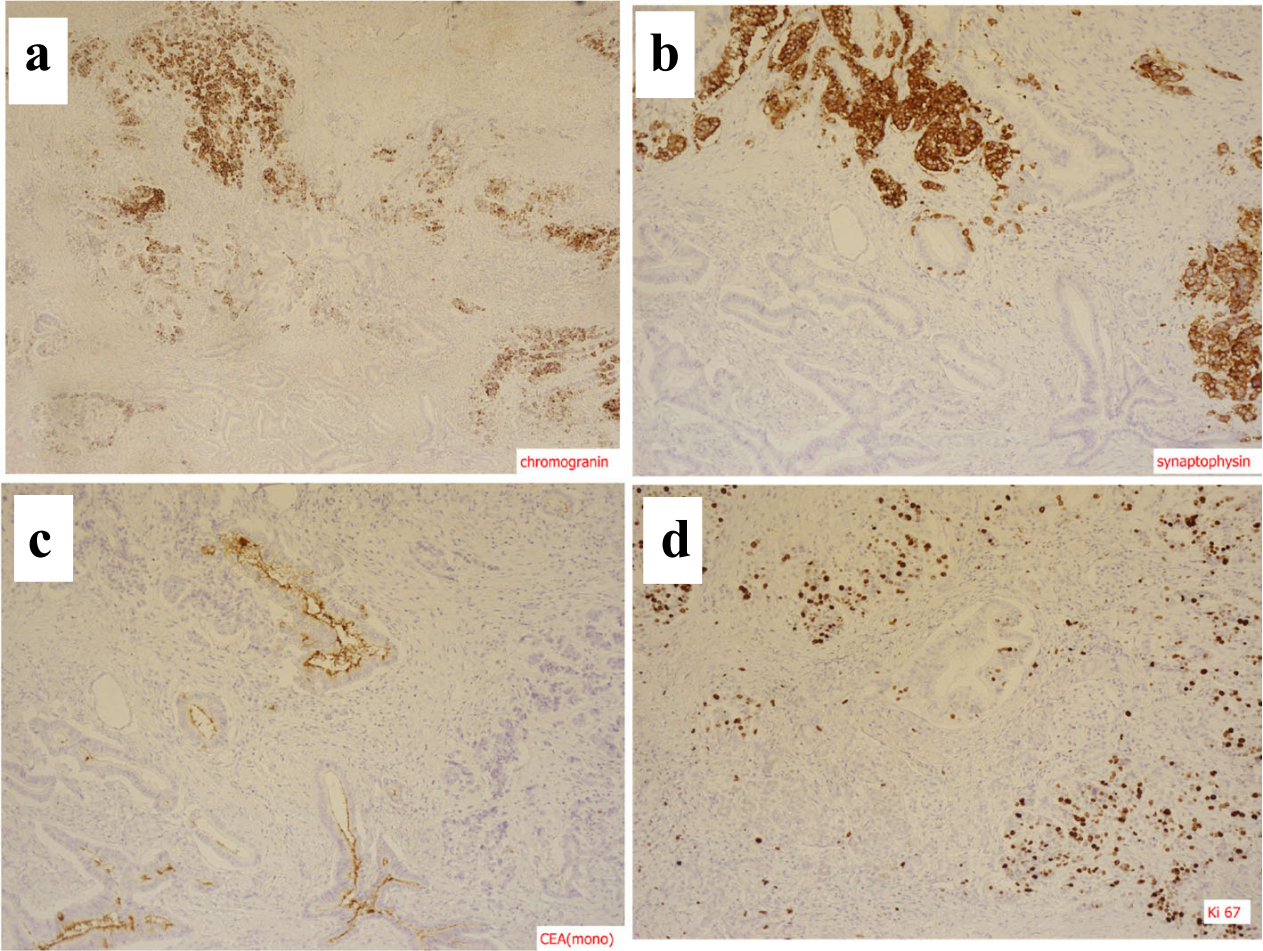
Immunohistochemically, the non-adenocarcinoma cell components were positive for the expression of CgA and Syn (**a, b**), but negative for CEA (**c**). The Ki67 labeling index was 40% (**d**)

Under the diagnosis of a neuroendocrine tumor, pancreaticoduodenectomy with portal vein resection and regional lymph node dissection was performed with curative intent. Macroscopically, a 2-cm-sized mass in the pancreas head was presented as the resected specimen. Histological examination revealed that the tumor consisted of two cell populations (Fig. 3). One was well- to moderately differentiated tubular adenocarcinoma. The adenocarcinoma cells were arranged in an irregular pattern and accounted for 45% of the whole tumor (Fig. 3b). The other cell population was non-adenocarcinoma cells arranged in a nest, and the cells had round nuclei, abundant cytoplasm, and coarse chromatin (Fig. 3c). An intermixed central zone exists between the two cell components (Fig. 3d). Immunohistochemically, the adenocarcinoma cells were positive for CEA but negative for chromogranin A (CgA) and synaptophysin (Syn), while the non-adenocarcinoma cells were positive for the expression of CgA and Syn but negative for CEA (Fig. 4a–c). The Ki67 labeling index was 40% (Fig. 4d). Based on these findings, a diagnosis of MANEC of the pancreas was made. The portal vein, duodenum, and peripheral lymph nodes were infiltrated by neuroendocrine carcinoma cells. The final pathological stage was T4 N2 M0 stage IVb according to the General Rules for the Study of Pancreatic Cancer in Japan [7].

The patient’s postoperative course was eventful, and he was discharged on the 34th day after the operation. He underwent adjuvant chemotherapy consisting of a combination irinotecan and cisplatin. However, he refused to continue chemotherapy after the completion of one course. Lymph node metastasis and peritoneal dissemination developed rapidly, and he died 6 months after the operation.

### Discussion

The term MANEC was introduced by the 2010 WHO classification of neuroendocrine neoplasms in the digestive system [1]. Neuroendocrine tumors are classed as NET G1 (carcinoid, mitotic count of <2 per 10 high power fields (HPF) and/or a Ki67 index of ≤2%); NET G2 (mitotic count of 2–20 per 10 HPF and/or a Ki67 index of 3–20%); and NET G3 (neuroendocrine carcinoma, mitotic count of >20 per 10 HPF and/or a Ki67 index of >20%). Furthermore, tumors with two malignant components, adenocarcinoma and neuroendocrine carcinoma, with each component accounting for at least 30% of the tumor, are defined as MANECs [1].

The pancreas is composed of exocrine and endocrine gland components. The exocrine part consists of ductal and acinar cells, and the endocrine part consists of islet cells. Most pancreatic tumors usually originate in one of these cell types. Pancreatic tumors containing both exocrine and endocrine components are rare. Cubilla and Fitzgerald reported that mixed cell type carcinomas accounted for only 0.2% of all pancreatic tumors [8]. These types of neoplasms are categorized as combined neoplasms of the pancreas in the General Rules for the Study of Pancreatic Cancer in Japan [7]. These tumors have been described by the terms “mixed duct-acinar tumors,” “duct-islet tumors,” “acinar-islet tumors,” and “duct-acinar-islet cell tumors,” based on their components. Among them, to the best of our knowledge, only four cases including our case are compatible with MANEC [9–11].

The clinicopathologic features of the four cases are summarized in Table 1. Three patients were males and one patient was female. The mean age of the patients was 62.5 years (range, 52–72 years). The mean size of the tumors was 2.1 cm (range, 2.0–2.5 cm). The locations were the pancreas body in three cases and the pancreas head in one case. Three of the four patients were asymptomatic. The tumors were incidentally detected by a CT scan during follow-up for other diseases. Generally, pancreatic neuroendocrine tumors are classified as either functional or non-functional depending on their ability to secrete biologically active hormones. Our patient presented with hyperglycemia, but he did not satisfy the criteria of glucagonoma syndrome. All cases including our case lacked distinct or functional hormones.

**Table 1:** Resected cases of MANEC of the pancreas

Case	Age, gender	Location, size	Operation	Type of combination	NEC component (%)	LN meta	Adjuvant chemotherapy	Recurrence	Prognosis (months)	Year, author
1	63, female	Body, 2 cm	DP	Collision	~40	NEC	S-1	(–)	Alive (8)	2010, Terashi [9]
2	52, male	Body, 2.5 cm	DP	Collision	30	ND	GEM	(–)	Alive (9)	2012, Watanabe [10]
3	72, male	Body, 2 cm	DP	Collision	30	(–)	S-1	(–)	Alive (8)	2015, Shibuya [11]
Our case	63, male	Head, 2 cm	PD	Collision	55	NEC	CPT-11 + CDDP	Peritoneal dissemination	Dead (6)	

*DP* distal pancreatectomy, *PD* pancreaticoduodenectomy, *NEC* neuroendocrine carcinoma, *GEM* gemcitabine, *CPT-11* irinotecan, *CDDP* cisplatin, *ND* not described

Radiological examinations are useful for the diagnosis of pancreatic tumors. Neuroendocrine tumors are typically well-circumscribed lesions that appear hyperenhancing on contrast-enhanced CT and show little change in the main pancreatic duct on endoscopic retrograde pancreatography (ERP). Narrowing or obstruction of the pancreatic duct is rare [12]. On the other hand, adenocarcinomas are typically hypovascular lesions on contrast-enhanced CT and show severe stenosis or obstruction in the main pancreatic duct on ERP. In cases of MANEC, radiological findings have both features of neuroendocrine carcinoma and adenocarcinoma. Imaging dynamics of contrast-enhanced CT vary depending on the ratios of neuroendocrine carcinoma cells and adenocarcinoma cells. Hence, diagnosis by means of only radiological examinations is difficult.

Recently, EUS-FNA has been reported to be a useful diagnostic tool for pancreatic tumors [13]. In case 3, MANEC was successfully diagnosed preoperatively by means of EUS-FNA. In our case, contrary to the final diagnosis, cytological analysis by EUS-FNA suggested a neuroendocrine tumor (G2). This may suggest that FNA has some limitations for making a definite diagnosis, because FNA cytology cannot cover the entire tumor. The confirmed diagnosis mainly depends on histopathological and immunohistochemical analyses from a surgically resected specimen. Furthermore, it is impossible to determine whether each component accounts for at least 30% if it cannot be resected completely.

The histogenesis of MANEC of the pancreas is still controversial. It was suggested in previous reports that associated exocrine and endocrine neoplasms of the pancreas arise from totipotent pancreatic stem cells, which reside in the pancreatic duct and islets [14, 15]. Therefore, tumors containing both exocrine and endocrine components can develop in the pancreas. Chang et al. proposed a classification system of tumor histogenesis for associated exocrine and endocrine neoplasms that consists of five types: amphicrine type, mixed type, collision type, solitary concomitant type, and multiple concomitant type [16]. Among them, the collision type shows the endocrine part at one end and the exocrine part at the other end, with an intermixed central zone. Our case can be classified as a collision type.

Due to the small number of cases of reported MANEC, the clinical behavior is unclear. At present, it is generally agreed that surgery is the first line of treatment for cases with a resectable tumor (Table 1). After radical resection, multimodal treatment with adjuvant radiotherapy and/or chemotherapy should be performed. However, it is still not clear whether the postoperative course and ideal management of cases of MANEC differ from those of cases of ductal adenocarcinoma only or neuroendocrine carcinoma only. Postoperative chemotherapies for ductal adenocarcinoma are now almost established [17, 18]. Also, in cases of neuroendocrine carcinoma, the efficacy of chemotherapeutic combination regimens such as etoposide plus cisplatin and irinotecan plus cisplatin has been reported [19, 20]. Lee et al. proposed that treatment should focus on the more aggressive cells of the tumor because the clinical outcome of this mixed tumor follows that of a more aggressive cell type [4]. In our case, the neuroendocrine carcinoma component mainly occupied the tumor and infiltrated the portal vein, duodenum, and peripheral lymph nodes, while the adenocarcinoma component was not seen in the infiltrated region. Accordingly, a combination regimen of irinotecan and cisplatin was administered to the patient. However, he refused to continue the chemotherapy, and lymph node metastasis and peritoneal dissemination developed rapidly. He died 6 months after the operation. Our case indicated that MANEC of the pancreas may be a tumor with high malignant potential. The long-term postoperative courses have not been fully described in reported cases, and it is therefore unclear whether the prognosis of MANEC is pessimistic or not. Further investigation of more cases of this rare entity is necessary.

## Conclusions

We have reported a case of MANEC of the pancreas. MANEC of the pancreas is extremely rare, and the clinical behavior remains unclear. Accumulation of additional data from more cases is necessary to further elucidate this type of tumor and standardize optimal therapy.

## Abbreviations

CA19-9: carbohydrate antigen 19-9
CEA: carcinoembryonic antigen
CgA: chromogranin A
CT: computed tomography
ERCP: endoscopic retrograde cholangiopancreatography
EUS-FNA: endoscopic ultrasonography-guided fine-needle aspiration
FDG-PET: fluorin-18 fluorodeoxyglucose-positron emission tomography
MANEC: mixed adenoneuroendocrine carcinoma
Syn: synaptophysin
